# Comparative outcomes of corticosteroids, neuromuscular blocking agents, and inhaled nitric oxide in ARDS: a systematic review and network meta-analysis

**DOI:** 10.3389/fmed.2025.1507805

**Published:** 2025-02-03

**Authors:** Zhiyuan Xu, Xiao Liu, Liang Zhang, Xianliang Yan

**Affiliations:** ^1^Department of Emergency Medicine, Xuzhou No. 1 People’s Hospital, Xuzhou, Jiangsu, China; ^2^Department of Emergency Medicine, Affiliated Hospital of Xuzhou Medical University, Xuzhou, Jiangsu, China; ^3^Department of Emergency Medicine, Suining County People’s Hospital, Xuzhou, Jiangsu, China

**Keywords:** ARDS, corticosteroids, NMBAs, iNO, network meta-analysis

## Abstract

**Objectives:**

Acute respiratory distress syndrome (ARDS) is associated with high rates of morbidity and mortality. However, the evidence regarding the effectiveness of commonly used treatments, including corticosteroids, neuromuscular blocking agents (NMBAs), and inhaled nitric oxide (iNO), remains uncertain. Therefore, this study aimed to compare and rank these three treatments to identify the most effective option.

**Data sources:**

We searched PubMed, Embase, Cochrane Library, and Web of Science for clinical trials from the earliest records to 1 May 2024.

**Study selection and data extraction:**

Clinical trials evaluating three interventions compared with the control group for ARDS were included, with restrictions on any language. Data were extracted by two independent reviewers. Frequentist network meta-analysis (NMA) was performed to identify the most effective intervention, and treatments were ranked using the surface under the cumulative ranking (SUCRA) curve. The primary outcome was 28-day mortality, while secondary outcomes included ventilator-free days up to 28 days, ICU mortality, in-hospital mortality, and the incidence of new infection events.

**Data synthesis:**

Data from 26 clinical trials encompassing 5,071 patients were analyzed. Vecuronium bromide was the most effective strategy for reducing 28-day mortality compared to conventional treatment, iNO, methylprednisolone, and placebo (OR 0.38, 95% CI 0.15–1.00, and OR 0.30, 95% CI 0.10–0.85 and OR 0.25, 95% CI 0.08–0.74 and OR 0.23, 95% CI 0.08–0.65; SUCRA: 96.6%). Dexamethasone was identified as the most effective treatment option for increasing ventilator-free days at 28 days compared to conventional therapy and cisatracurium (MD 3.60, 95% CI 1.77–5.43, and MD 3.40, 95% CI 0.87–5.92; SUCRA: 93.2%). Methylprednisolone demonstrated the highest effectiveness for preventing ICU mortality (SUCRA: 88.5%). Although dexamethasone, cisatracurium, conventional therapy, methylprednisolone, and iNO treatment did not show significant superiority in reducing in-hospital mortality, dexamethasone showed the highest probability of being the most effective treatment option (SUCRA: 79.7%). Furthermore, dexamethasone treatment showed the highest safety in reducing the incidence of new infection events compared with placebo and iNO (OR 0.61, 95% CI 0.42–0.88, and OR 0.33, 95% CI 0.19–0.58; SUCRA: 91.8%).

**Conclusion:**

This NMA suggests that corticosteroids may provide benefits to patients with ARDS. While the application of NMBAs may reduce 28-day mortality, iNO did not demonstrate a significant beneficial effect as a therapeutic measure.

**Systematic review registration:**

PROSPERO, CRD42022333165 https://www.crd.york.ac.uk/PROSPERO/.

## Introduction

Acute respiratory distress syndrome (ARDS) is a severe global health issue characterized by high morbidity and mortality. It is defined as chronic respiratory failure caused by non-hemodynamic pulmonary edema due to inflammatory cytokines (1, 2). The COVID-19 pandemic has further underscored the severity of ARDS, increased its incidence, and revealed the critical need for effective treatments (3). Globally, ARDS affects 10.4% of intensive care unit patients and 23.4% of intubated patients, with an associated hospital mortality rate of 40% (2). Since its initial description in 1967 (4), numerous management strategies for ARDS have been clinically evaluated (5, 6). Despite over 50 years of research, none of the available treatments directly target the pathophysiological mechanisms underlying acute respiratory failure (7).

Recent guidelines on ARDS management (8), developed following the National Institute for Health and Care Excellence (NICE) methodology, emphasize evidence-based interventions. Based on existing recommendations and expert consensus, corticosteroids, neuromuscular blocking agents (NMBAs), and inhaled nitric oxide (iNO) were identified as key focus areas. Corticosteroids target the inflammatory cascade central to ARDS pathophysiology with their anti-inflammatory and antifibrotic properties. NMBAs improve oxygenation and reduce ventilator-induced lung injury by enhancing patient–ventilator synchrony. At the same time, iNO, a selective pulmonary vasodilator, is often used to improve oxygenation in severe cases despite its controversial efficacy. Although these treatments are widely utilized, systematic reviews and meta-analyses (SR/MAs) have produced mixed results regarding their effectiveness and safety. Some evidence suggests that corticosteroids reduce mortality and increase ventilator-free days (VFD) (9), while other analyses indicate that early and prolonged corticosteroid use may further improve outcomes (10). Studies show potential benefits of NMBAs, including improved oxygenation, reduced ventilator-induced lung injury, and decreased 28-day mortality rates (11). The role of iNO in ARDS management has been evaluated through multiple randomized controlled trials (RCTs) and subsequent SR/MAs, though its efficacy remains debated.

The COVID-19 pandemic has amplified the global relevance of ARDS, with the SARS-CoV-2 outbreak driving a sharp increase in ARDS-related mortality (3). This highlights the urgent need to synthesize existing evidence to inform current critical care practices. We conducted a network meta-analysis (NMA) of high-quality trials to evaluate the efficacy of corticosteroids, NMBAs, and iNO in ARDS management, with the goal of informing treatment strategies and improving patient outcomes.

## Materials and methods

### Literature review

We conducted a systematic search of PubMed, Embase, Cochrane Library, and Web of Science from their inception to 1 May 2024. Details of the search strategy are provided in Appendix 1. This meta-analysis adhered to guidelines outlined in the Preferred Reporting Items for Systematic Reviews and Meta-Analyses (PRISMA) and was registered on PROSPERO (CRD42022333165).

### Inclusion and exclusion criteria

Eligible studies met the following inclusion criteria: (I) adult patients (aged ≥18 years) who had ARDS treated with the three interventions, as outlined in the 2019 Guidelines on the Management of ARDS (8); (II) interventions involving corticosteroids, iNO, or NMBAs in the treatment group, with a matched placebo or conventional therapy in the control group. Studies were excluded if they met any of the following criteria: (I) publication types such as letters, case reports, reviews, or editorials; (II) *in vitro* or animal studies; (III) insufficient or unavailable data. No language restrictions were applied.

### Data collection and outcomes

Two reviewers (Zhiyuan Xu and Xiao Liu) independently extracted data using a standardized form. Any disagreements were resolved through consensus or, when necessary, a third reviewer (Pro Yan). The primary outcome was 28-day mortality, defined as the mortality rate on the 28th day of treatment. The secondary outcomes included ventilator-free days at 28 days (the number of days without ventilator use by day 28), ICU mortality, in-hospital mortality, and the occurrence of new infections.

### Risk of bias assessment and quality assessment

Two reviewers (Zhiyuan Xu and Liang Zhang) independently screened articles and assessed studies for inclusion. Discrepancies were resolved through discussion with a third reviewer (Pro Yan). The reviewers evaluated the risk of bias using the Cochrane assessment tool, considering seven domains such as random sequence generation, allocation concealment, blinding of participants and personnel, blinding of outcome assessment, incomplete outcome data, selective reporting, and other potential biases (12). Each domain was graded as low, high, or unclear risk of bias. Any disagreements regarding the quality assessment were resolved through consensus with the third reviewer.

### Statistical analysis

We performed a frequentist NMA using the mvmeta command in Stata 16.0. All treatment comparisons were presented using a network graph for each outcome. The nodes in the evidence diagram represented different intervention measures, and the lines between these nodes represented different head-to-head comparisons. Dichotomous variables were summarized as odds ratios (ORs) with 95% confidence intervals (CIs), while continuous variables were expressed as mean differences (MDs) with 95% CIs. Statistical heterogeneity was initially assessed in each comparison using the *I^2^* statistic, which categorizes heterogeneity as low (<25%), moderate (25–50%), or high (>50%) (13).

To provide a more comprehensive assessment of heterogeneity, we also calculated τ^2^ values, which were categorized based on established guidelines as low (<0.04), low-to-moderate (0.04–0.16), moderate-to-high (0.16–0.36), and high (>0.36). Model selection was guided by heterogeneity measures: a random-effects model was adopted when *I^2^* > 50% or when τ^2^ values suggested notable heterogeneity; otherwise, a fixed-effects model was applied (14). Residual deviance was assessed using a chi-squared (chi^2^) test to evaluate the model adequacy. The residual deviance was compared to its degrees of freedom (df), with values closer to the df indicating a better model fit.

We presented rank probabilities for each intervention and determined the treatment hierarchy using the surface under the cumulative ranking curve (SUCRA) (15). Higher SUCRA probabilities indicate a higher likelihood of being the most effective treatment. Ranking results were interpreted in the context of relative risk estimates and their corresponding 95% CIs for each comparison. To assess the robustness of our findings, sensitivity analyses were conducted to evaluate the impact of risk of bias on the overall study outcomes. We visually inspected funnel plots for publication bias, focusing on primary outcomes and adverse events. No substantial asymmetry was detected, suggesting minimal risk of publication bias.

## Results

### Characteristics, risk of bias, and consistency

**Figure 1:**
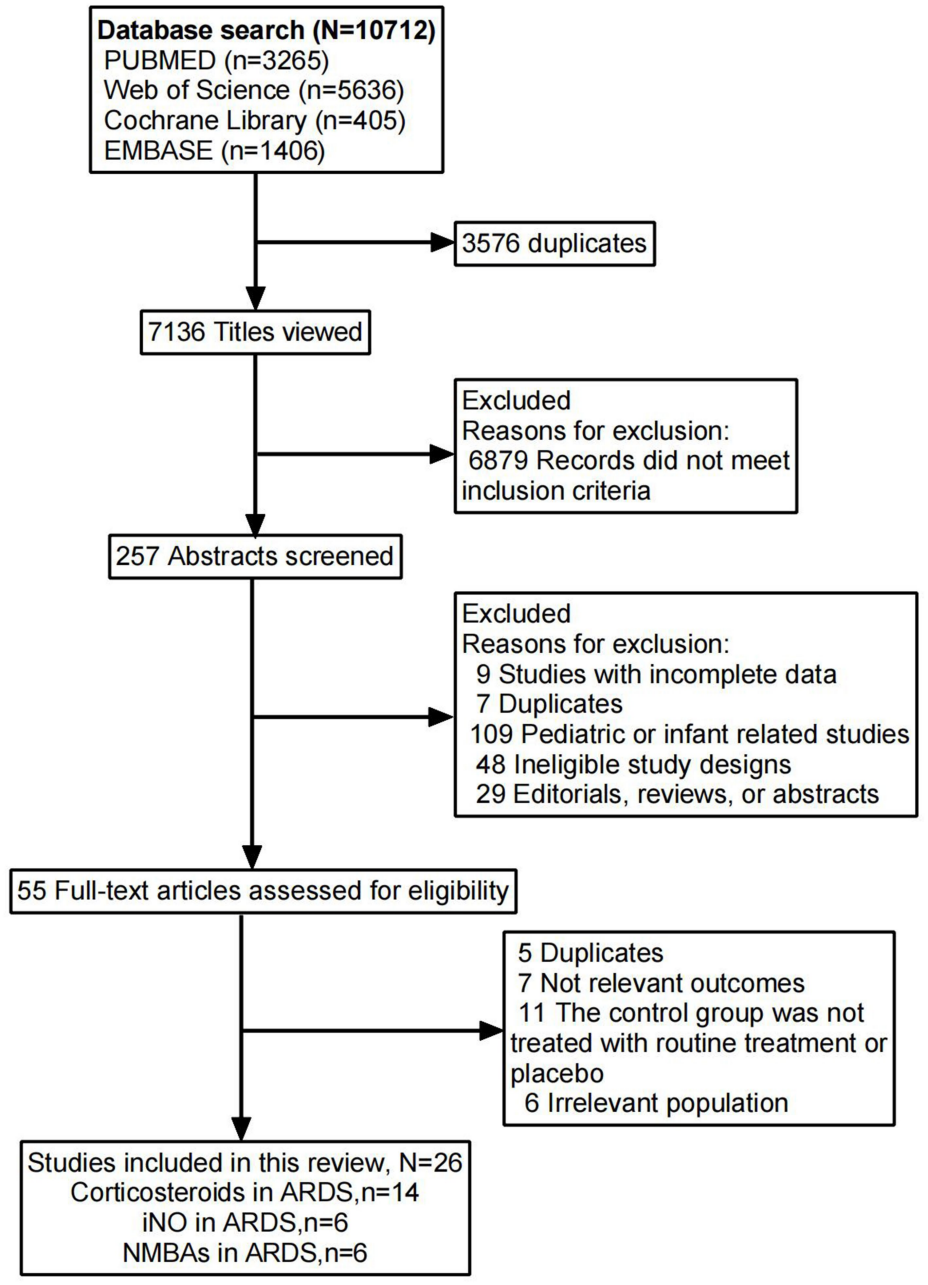
Flowchart for identifying studies eligible for the meta-analysis.

The risk of bias assessments for the studies included in each outcome analysis are detailed in Supplementary Figure 1. A total of 7,136 reports were screened, of which 26 trials involving 5,071 participants met the eligibility criteria (Figure 1). These trials comprised 24 studies published in English (16–39) and 2 high-quality studies published in Chinese (40, 41).

Among the 26 included studies, 12 (16, 17, 20, 22, 24, 26, 29–31, 33, 34, 37) reported ventilator-free days at 28 days, and 17 (18, 20–22, 26, 27, 29, 30, 32–34, 36–41) provided data on 28-day mortality. ICU mortality was assessed in 10 studies (16, 20, 21, 24, 25, 30–33, 35), while 14 studies (16, 18–20, 23–25, 28–30, 32, 33, 35, 36) reported in-hospital mortality. Additionally, 13 studies (16, 17, 19–21, 24–28, 36, 37, 40) provided data on new infection events. The baseline characteristics of all included studies are summarized in Table 1.

**Table 1:** Characteristics of the included studies.

Study	Year	Country	Research type	Sample size	Treatment group (PaO2/FiO_2_, mm Hg)	Control group (PaO2/FiO_2_, mm Hg)	Primary outcome	Secondary outcome	Score of NOS	Score of Jadad
Villar (16)	2020	Spain	Randomized controlled trial	277	142.4 ± 37·3	143.5 ± 33.4	Ventilator-free days at 28 days	All-cause mortality 60 days	–	5
Steinberg (17)	2006	USA	Randomized controlled trial	180	126 ± 42	126 ± 40	Mortality at 60 days	Ventilator-free days at 28 days	–	5
Liu J (18)	2020	China	Retrospective study	774	168 (IQR 99–237)	168 (IQR 99–237)	28-day all-cause mortality.	In-hospital mortality	8	–
Bernard (19)	1987	USA	Randomized controlled trial	99	–	–	Mortality at 45 days	New infection events	–	5
Annane (20)	2006	France	Retrospective study	177	104 ± 42	108 ± 45	Ventilator-free days at 28 days	Mortality in ICU	7	–
Meduri (21)	2018	USA	Retrospective study	180	–	–	Mortality at 28 days	New infection events	8	–
Tongyoo (22)	2016	Thailand	Randomized controlled trial	197	175.4 ± 6.9	172.4 ± 6.7	28-day all-cause mortality	Ventilator-free days at 28 days	–	5
HS Lee (23)	2005	Korea	Retrospective study	20	142.5 ± 23.7	143.4 ± 23.9	In-hospital mortality	Hospital stay days	7	–
Meduri (24)	2007	USA	Randomized controlled trial	91	118.4 ± 51.2	125.9 ± 38.6	Ventilator-free days at 28 days	Mortality in ICU	–	4
Meduri (25)	1998	USA	Randomized controlled trial	24	161 ± 14	141 ± 19	Lung function and mortality	MODS scores	–	5
Tomazini (26)	2020	Brazil	Randomized controlled trial	299	131.1 ± 46.2	132.6 ± 45.7	Ventilator-free days at 28 days	All-cause mortality at 28 days	–	5
Liu L (40)	2012	China	Randomized controlled trial	26	138.2 (87.0, 171.0)	157.0 (88.7, 176.3)	Mortality at 28 days	New infection events	–	4
Varpula (27)	2000	Finland	Retrospective study	31	126.3 ± 52.4	107 ± 41.4	Mortality at 28 days	New infection events	7	–
Buisson (28)	2011	French	Retrospective study	208	101 (73–174)	107 (78–144)	In-hospital mortality	New infection events	9	–
Moss (29)	2019	USA	Randomized controlled trial	1,006	98.7 ± 27.9	99.5 ± 27.9	In-hospital mortality	Organ dysfunction	–	5
Gainnier (30)	2004	France	Randomized controlled trial	56	130 ± 34	119 ± 31	Ventilator-free days at 28 days	Mortality in ICU	–	4
Guervilly (31)	2016	France	Randomized controlled trial	24	158 (131; 185)	150 (121; 187)	Ventilator-free days at 28 days	Mortality in ICU	–	5
Forel (32)	2006	France	Randomized controlled trial	36	105 ± 22	125 ± 20	Mortality at 28 days	Mortality in ICU	–	4
Papazian (33)	2010	France	Randomized controlled trial	340	106.0 ± 36.0	115.0 ± 41.0	The 90-day mortality	The day-28 mortality	–	5
Lyu (41)	2014	China	Randomized controlled trial	96	141.0 ± 26.1	144.3 ± 24.1	Mortality at 28 days	APACHE 1I scores	–	4
Dellinger (34)	1998	USA	Randomized controlled trial	177	135 ± 41.0	129.0 ± 38.0	Ventilator-free days at 28 days	The day-28 mortality	–	4
Gerlach (35)	2003	Germany	Randomized controlled trial	40	113.0 ± 28.0	104.0 ± 26.0	Duration of ventilation	Mortality in ICU	–	4
Lundin (36)	1999	UK	Randomized controlled trial	268	102.8 ± 32.3	100.5 ± 33.0	The day-28 mortality	New infection events	–	4
Taylor (37)	2004	USA	Randomized controlled trial	385	133.0 ± 42.0	138.0 ± 43.0	Ventilator-free days at 28 days	The day-28 mortality	–	5
Troncy (38)	1998	Canada	Randomized controlled trial	30	–	–	The day-28 mortality	APACHE II scores	–	4
Cuthbertson (39)	2000	UK	Randomized controlled trial	30	–	–	The day-28 mortality	APACHE II scores		4

The consistency analyses for all outcomes showed *p*-values greater than 0.05, indicating good consistency (Supplementary Table 1). Additionally, only two outcomes, in-hospital mortality and new infection events, formed closed loops, allowing for further node-splitting analysis. The results of the node-splitting method also indicated *p*-values greater than 0.05, confirming good consistency (Supplementary Tables 2, 3). Heterogeneity, assessed using τ^2^, suggested that overall heterogeneity was low to moderate across all outcomes (Supplementary Table 1). Furthermore, funnel plots showed good symmetry, indicating no significant publication bias (Supplementary Figure 2).

### 28-day mortality

**Figure 2:**
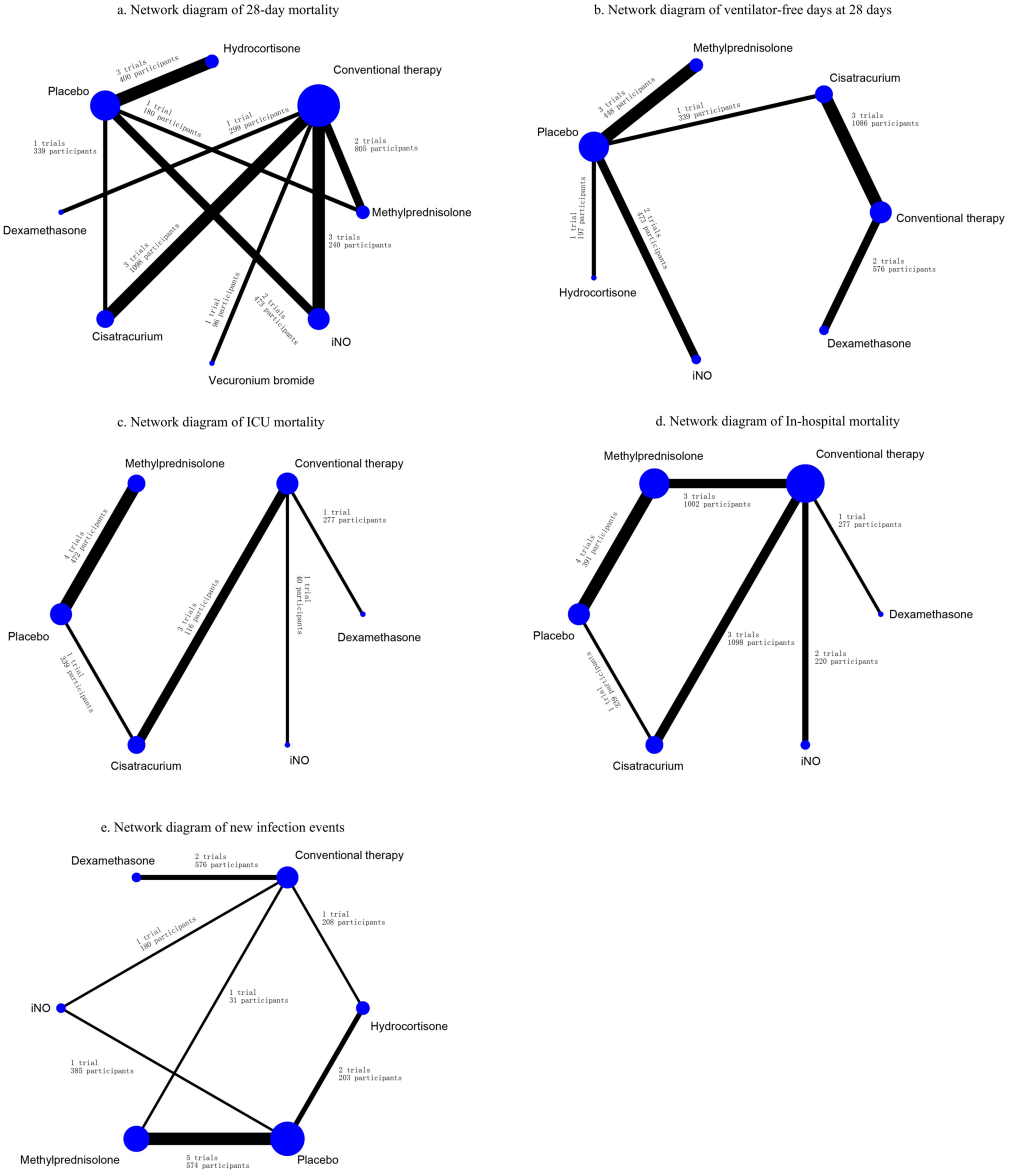
Network diagrams for the association between interventions and outcomes. **(A)** Network diagram of 28-day mortality. **(B)** Network diagram of ventilator-free days at 28 days. **(C)** Network diagram of ICU mortality. **(D)** Network diagram of in-hospital mortality. **(E)** Network diagram of new infection events. Network diagrams showing ARDS treatment comparisons in clinical trials with respect to the number of studies and sample sizes. The width of the line is proportional to the number of trials directly comparing each pair of treatments, and the size of each node is proportional to the sample size of randomized participants.

**Figure 3:**
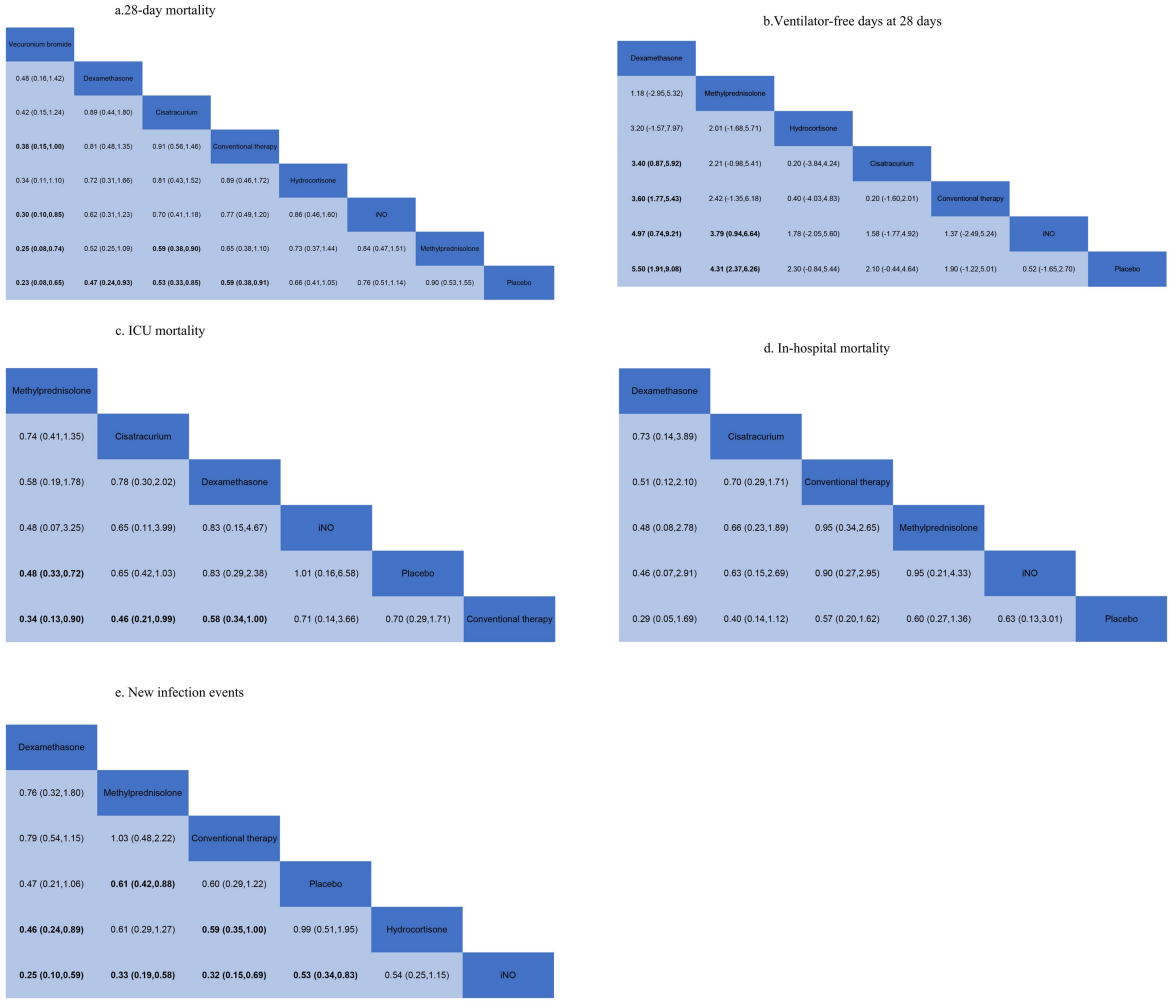
Summary of results from network meta-analysis. **(A)** 28-day mortality. **(B)** Ventilator-free days at 28 days. **(C)** ICU mortality. **(D)** In-hospital mortality. **(E)** New infection events.

**Figure 4:**
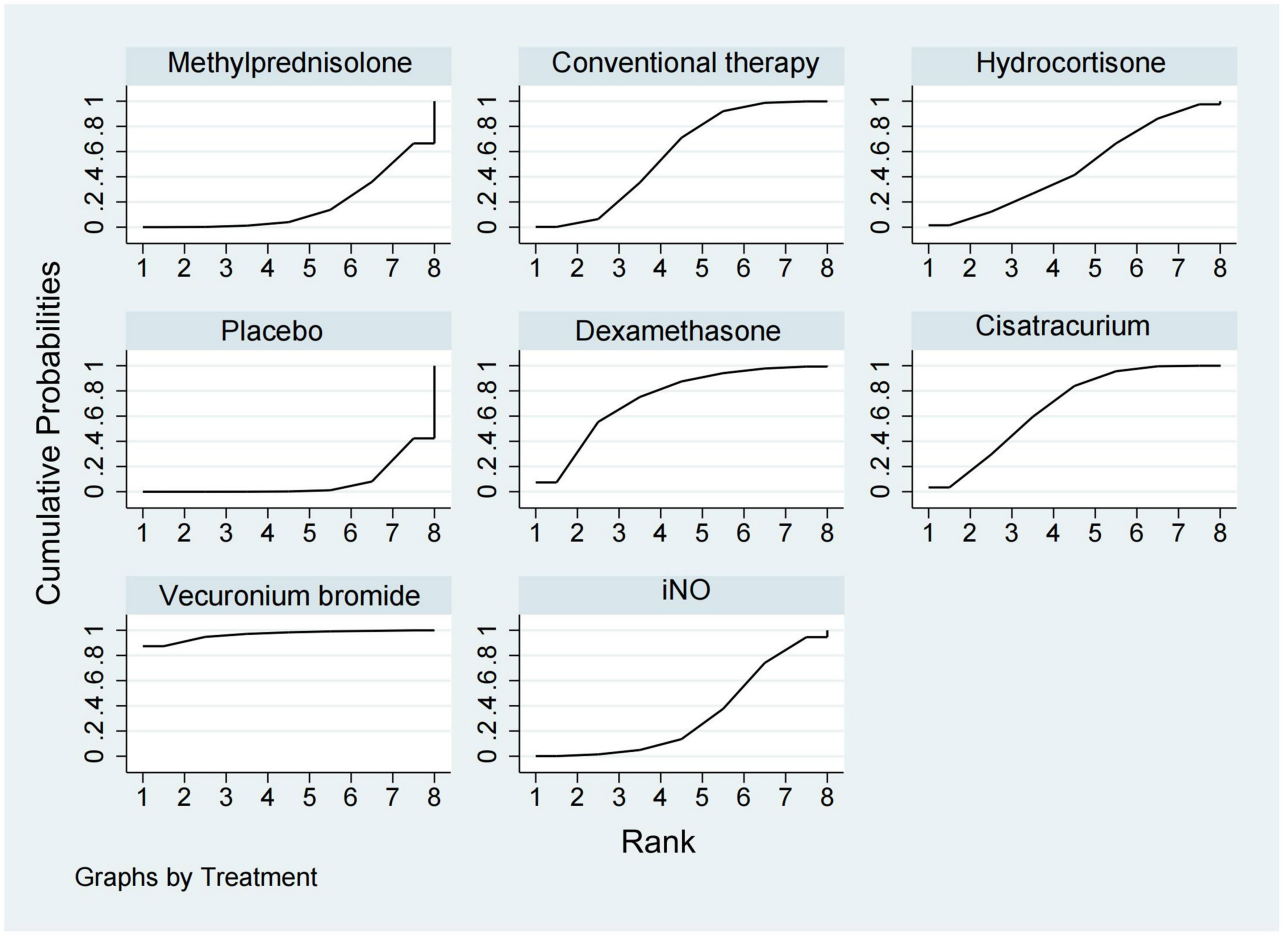
Ranking of treatment strategies based on the probability of their protective effects on outcomes of 28-day mortality according to the cumulative ranking area (SUCRA).

Data regarding the efficiency of corticosteroids, iNO, and NMBAs on 28-day mortality were available from 17 trials (18, 20–22, 26, 27, 29, 30, 32–34, 36–41) involving 3,930 patients. As shown in Figures 2, 3, vecuronium bromide was more effective than conventional therapy (OR 0.38, 95% CI 0.15–1.00), iNO (OR 0.30, 95% CI 0.10–0.85), methylprednisolone (OR 0.25, 95% CI 0.08–0.74), and placebo (OR 0.23, 95% CI 0.08–0.65). Dexamethasone was only better than placebo (OR 0.47, 95% CI 0.24–0.93). Cisatracurium was found to be superior to methylprednisolone (OR 0.59, 95% CI 0.38–0.90) and placebo (OR 0.53, 95% CI 0.33–0.85). Conventional therapy also showed an advantage over placebo (OR 0.59, 95% CI 0.38–0.91). However, no treatment has shown significant advantages over the others when comparing hydrocortisone, iNO, methylprednisolone, and placebo. The effects of all drugs were ranked based on SUCRA probabilities (Figure 4). Vecuronium bromide had the greatest probability (SUCRA 96.6%) of being the best treatment option for reducing 28-day mortality in patients with ARDS, followed by dexamethasone (SUCRA 73.8%), cisatracurium (SUCRA 67.3%), conventional therapy (SUCRA 57.7%), hydrocortisone (SUCRA 47.4%), iNO (SUCRA 32.4%), methylprednisolone (SUCRA 17.4%), and placebo, which ranked last (SUCRA 7.4%).

### Ventilator-free days at 28 days

**Figure 5:**
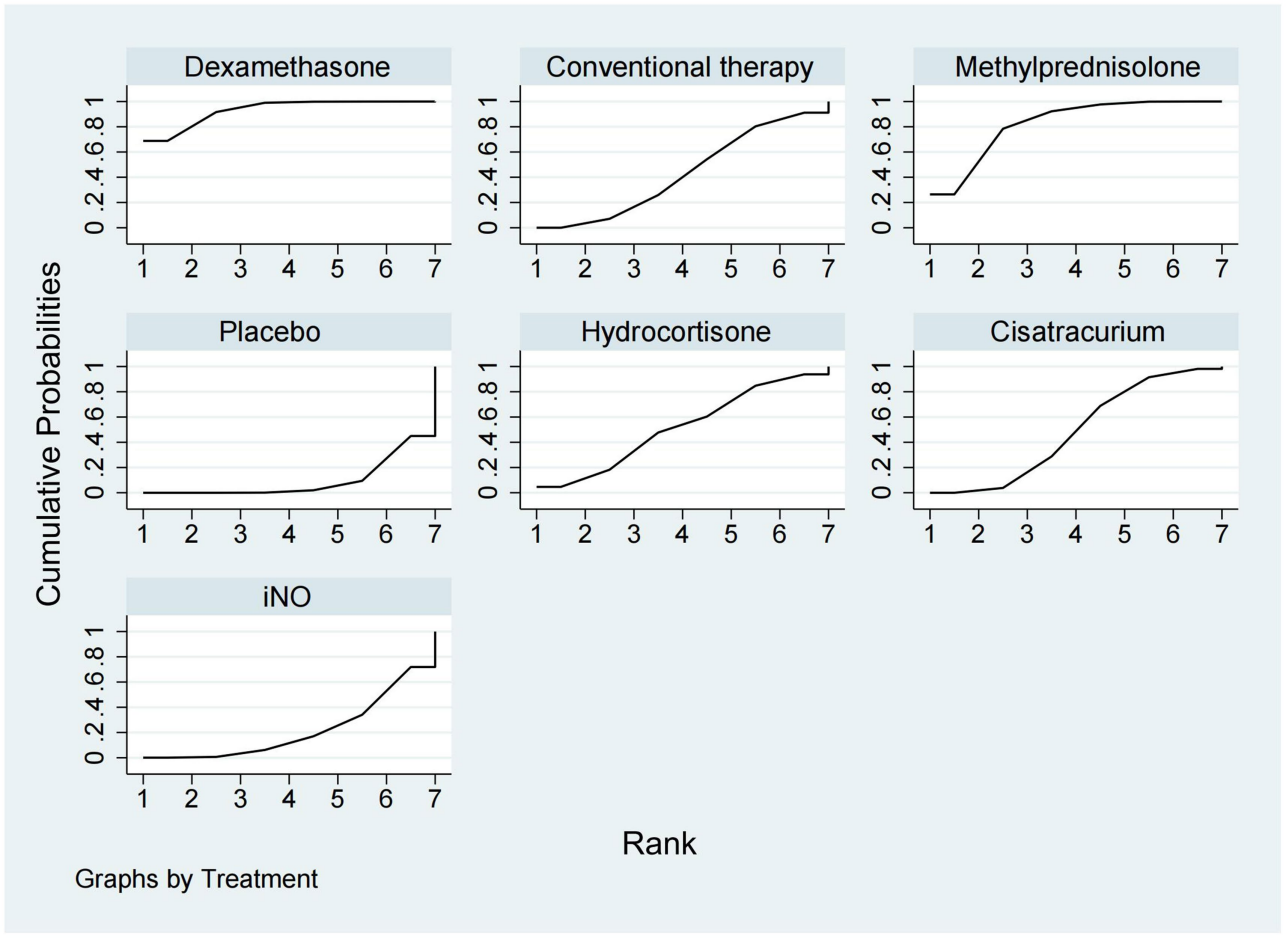
Ranking of treatment strategies based on the probability of their protective effects on outcomes of ventilator-free days at 28 days according to the surface under the cumulative ranking area (SUCRA).

A total of 12 studies (16, 17, 20, 22, 24, 26, 29–31, 33, 34, 37) involving 3,119 patients evaluated the effect of these drugs on ventilator-free days at 28 days. Compared to placebo, both dexamethasone and methylprednisolone increased ventilator-free days at 28 days (MD 5.50, 95% CI 1.91–9.08 and MD 4.31, 95% CI 2.37–6.26) (Figures 2, 3). Dexamethasone and methylprednisolone also have a significant benefit for ventilator-free days at 28 days (MD 4.97, 95% CI 0.74–9.21) and (MD 3.79, 95% CI 0.94–6.64) compared to the iNO group. Compared to conventional therapy and cisatracurium, dexamethasone showed a significant superiority (MD 3.60, 95% CI 1.77–5.43, and MD 3.40, 95% CI 0.87–5.92) in ventilator-free days at 28 days. However, iNO, cisatracurium, and hydrocortisone exhibited no superiority of ventilator-free days at 28 days over placebo or conventional therapy. As shown in Figure 5, dexamethasone had the highest probability of being the best treatment option for increasing ventilator-free days at 28 days (SUCRA 93.2%), followed by methylprednisolone (SUCRA 82.4%). Hydrocortisone (SUCRA 51.6%) and cisatracurium (SUCRA 48.5%) ranked third and fourth, respectively, followed by conventional therapy (SUCRA 43.1%), iNO (SUCRA 21.7%), and placebo (SUCRA 9.4%).

### ICU mortality

**Figure 6:**
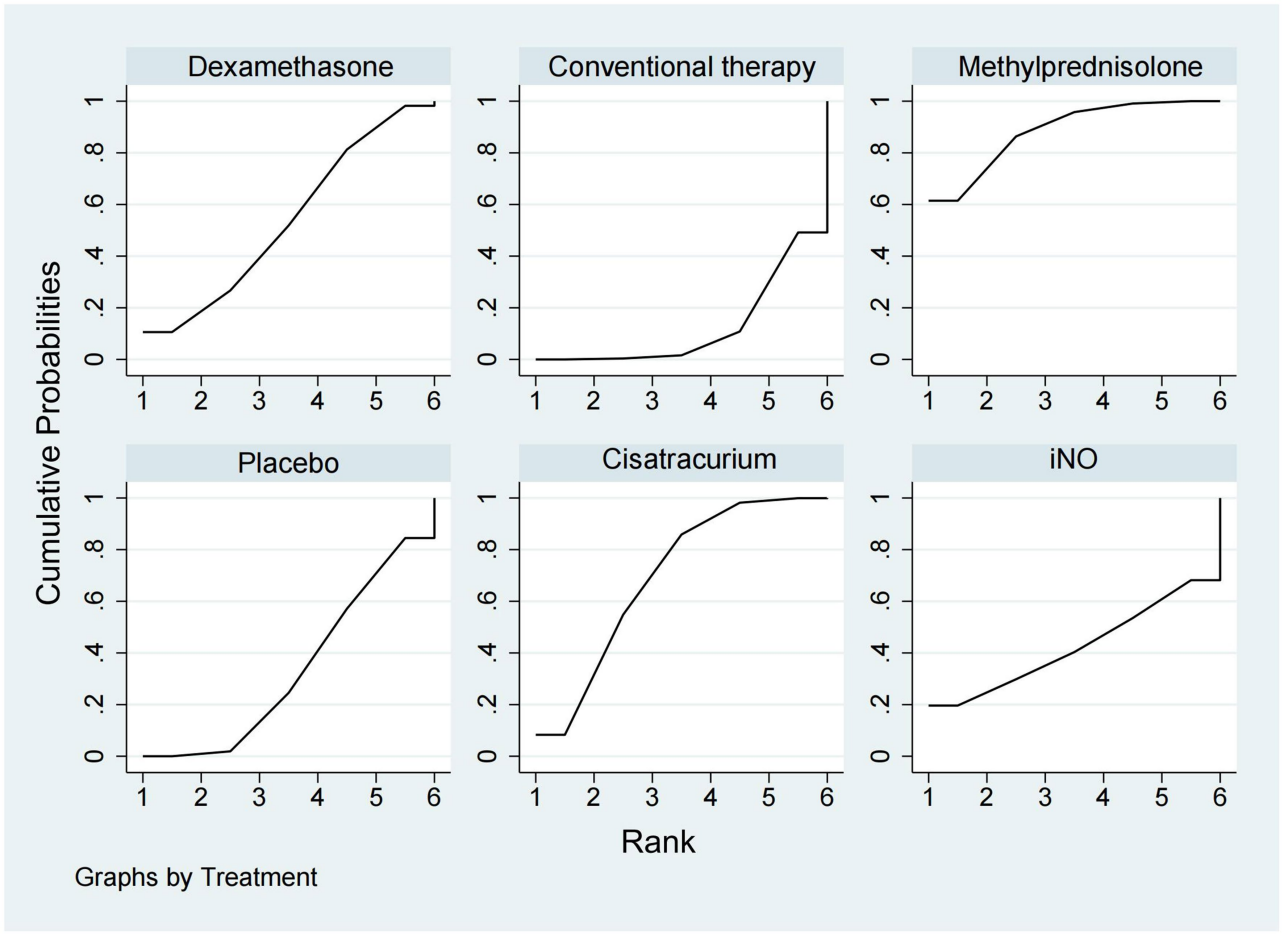
Ranking of treatment strategies based on the probability of their protective effects on outcomes of ICU mortality according to the surface under the cumulative ranking area (SUCRA).

ICU mortality was reported from a total of 10 studies (16, 20, 21, 24, 25, 30–33, 35) of 1,244 patients. Methylprednisolone significantly decreased the mortality in ICU compared with placebo (OR 0.48, 95% CI 0.33–0.72), as shown in Figures 2, 3. The therapy of methylprednisolone, cisatracurium, and dexamethasone was superior to conventional therapy in reducing ICU mortality (OR 0.34, 95% CI 0.13–0.90; OR 0.46, 95% CI 0.21–0.99; and OR 0.58, 95% CI 0.34–1.00). The therapy of iNO had no advantages in reducing mortality in the ICU over other treatments. Our results (Figure 6) suggested that, regarding prevention of ICU mortality, methylprednisolone (SUCRA 88.5%) was most effective, followed by cisatracurium (SUCRA 69.4%), dexamethasone (SUCRA 53.7%), iNO (SUCRA 42.3%), placebo (SUCRA 33.6%), and conventional therapy (SUCRA 12.4%).

### In-hospital mortality

**Figure 7:**
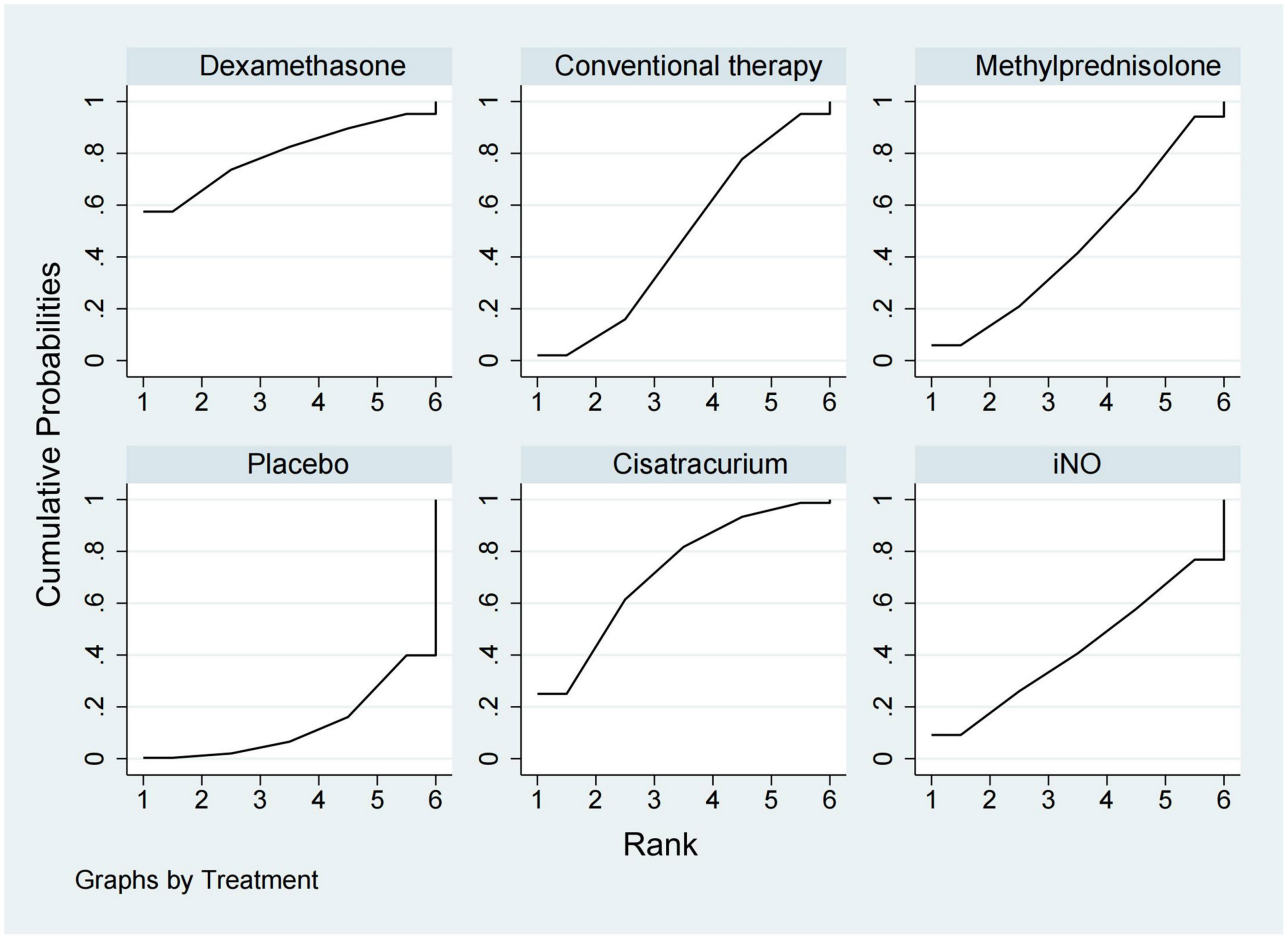
Ranking of treatment strategies based on the probability of their protective effects on outcomes of in-hospital mortality according to the surface under the cumulative ranking area (SUCRA).

A total of 14 studies (16, 18–20, 23–25, 28–30, 32, 33, 35, 36) involving 3,327 participants assessed in-hospital mortality. As shown in Figures 2, 3, compared with the conventional treatment or placebo, dexamethasone, cisatracurium, methylprednisolone, and iNO showed no significant advantages in reducing hospital mortality. As shown in Figure 7, dexamethasone reduced the incidence of in-hospital mortality at the top-ranking position (SUCRA 79.7%), followed by cisatracurium (SUCRA 72.1%), conventional therapy (SUCRA 47.6%), methylprednisolone (SUCRA 45.6%), iNO (SUCRA 42.1%), and placebo (SUCRA 13.0%).

### New infection events

**Figure 8:**
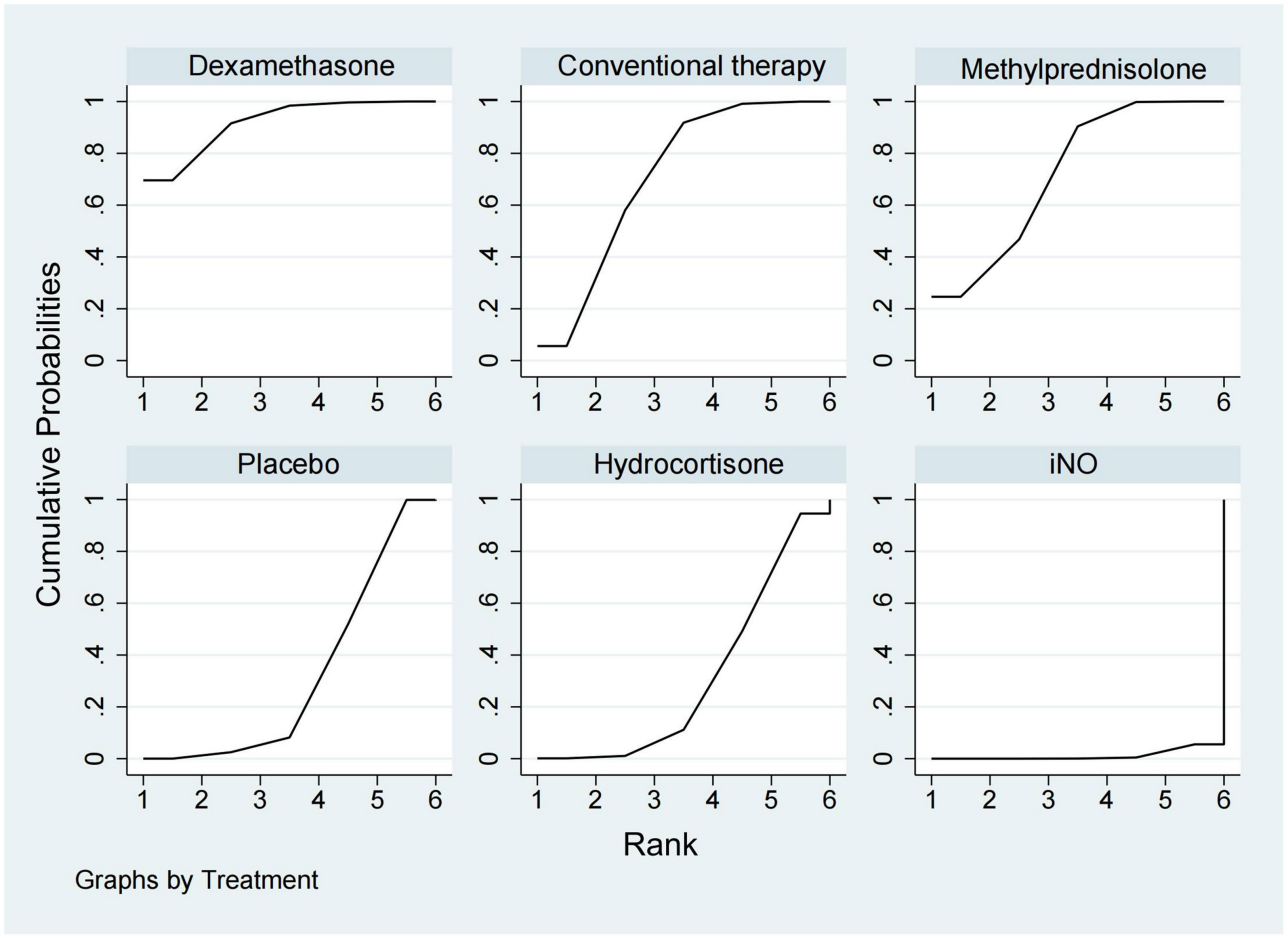
Ranking of treatment strategies based on the probability of their protective effects on outcomes of new infection events according to the surface under the cumulative ranking area (SUCRA).

Data regarding new infection events were available from 13 trials (16, 17, 19–21, 24–28, 36, 37, 40) involving 2,157 patients. Dexamethasone significantly decreased the rate of new infection events compared to hydrocortisone and iNO (OR 0.46, 95% CI 0.24–0.89, and OR 0.25, 95% CI 0.10–0.59), as shown in Figures 2, 3. Methylprednisolone had advantages in protecting against new infection events compared to placebo and iNO (OR 0.61, 95% CI 0.42–0.88 and OR 0.33, 95% CI 0.19–0.58). Conventional therapy also significantly reduced new infection events (OR 0.59, 95% CI 0.35–1.00 and OR 0.32, 95% CI 0.15–0.69) compared to hydrocortisone and iNO. In addition, placebo significantly decreased the rate of new infection events compared to iNO (OR 0.53, 95% CI 0.34–0.83). For decreasing the incidence of new infection events, dexamethasone showed the highest safety ranking (Figure 8, SUCRA 91.8%), followed by methylprednisolone (SUCRA 72.3%) and conventional therapy (SUCRA 70.9%). Placebo (SUCRA 32.5%) and hydrocortisone (SUCRA 31.2%) ranked fourth and fifth, respectively, while iNO ranked last (SUCRA 1.2%).

## Discussion

This is the first NMA to directly compare the efficacy and safety of corticosteroids, iNO, and NMBAs in adult patients with ARDS. Our findings suggest that vecuronium may be the most effective treatment for reducing 28-day mortality in these patients. However, its effect was not significantly different from dexamethasone, cisatracurium, or hydrocortisone. Dexamethasone and methylprednisolone demonstrated significant benefits in increasing ventilator-free days at 28 days. Additionally, methylprednisolone, cisatracurium, and dexamethasone showed significant efficacy in reducing ICU mortality, with methylprednisolone ranking highest. Dexamethasone and methylprednisolone were also effective in preventing new infections in ARDS patients. However, no intervention demonstrated superiority over others in reducing in-hospital mortality.

While our findings provide important insights, the applicability of these treatments may vary across different patient subgroups. ARDS is a highly heterogeneous syndrome with diverse causes, such as sepsis, trauma, and pneumonia, which may influence treatment response. For instance, corticosteroids may be more effective in treating ARDS caused by systemic inflammation (e.g., sepsis), given their anti-inflammatory and antifibrotic properties. In contrast, their benefits might be less pronounced in trauma-induced ARDS due to differences in inflammatory profiles. Similarly, age and comorbidities could impact treatment efficacy. Younger patients with greater physiological resilience may respond better to corticosteroids or NMBAs, while older patients or those with significant comorbidities, such as chronic kidney disease or diabetes, may be more susceptible to adverse effects, such as infections or myopathy. Future studies should explore these subgroups to further refine treatment recommendations.

Two recent studies (10) demonstrated that corticosteroids may reduce short-term mortality and the duration of mechanical ventilation in patients with ARDS. Corticosteroids, with their anti-inflammatory properties, help maintain endothelial integrity, reduce pro-inflammatory cytokines, and inhibit nitric oxide synthases, making them effective in managing various inflammatory conditions (10). Additionally, their antifibrotic properties, achieved by inhibiting fibroblast growth and collagen deposition, further support their therapeutic role (42). Another study (43) suggested that the effectiveness of corticosteroids in ARDS may vary depending on the specific medication used, a finding consistent with our results. The long-standing hypothesis that corticosteroid therapy benefits ARDS patients (17, 25) is strongly supported by the findings of our NMA. Our analysis confirmed that corticosteroids reduce short-term mortality and the duration of mechanical ventilation in ARDS patients. These results suggest that clinicians can consider corticosteroids to mitigate the immediate life-threatening risks associated with ARDS and to reduce the incidence of infections. Additionally, corticosteroids reduce ventilator dependency, promote the recovery of spontaneous breathing, and improve lung function, thereby contributing to overall patient recovery.

Muscle relaxation therapy, a common non-ventilatory strategy, is frequently employed by clinicians to treat moderate-to-severe ARDS (33). The benefits of NMBAs are primarily attributed to their pharmacological ability to control tidal volume and improve ventilator synchronization (44). NMBAs enhance patient–ventilator coordination by relaxing skeletal muscles and enabling better tidal volume management (45). The efficacy of NMBAs in ARDS is well documented. Numerous studies indicate that NMBAs can reduce ventilator-induced lung injury by improving man–machine synchronization, lowering oxygen consumption, and potentially exerting indirect anti-inflammatory effects. Over the past two decades, multiple clinical trials have evaluated the role of NMBAs in ARDS management (30–33, 41, 46). However, the results have often been inconsistent, complicating clinical decisions regarding their use. Our study provides new insights showing that NMBAs effectively reduce the 28-day mortality and ICU mortality of ARDS patients. These findings are more robust and innovative than those of some previous studies (11, 47–50), offering valuable evidence to guide clinical judgment on using NMBAs in ARDS management.

Nitric oxide was first identified as an endogenous vasodilator in 1987 (51), leading to its application in treating pulmonary hypertension and lung diseases (52, 53). Currently, iNO is commonly used to manage pulmonary hypertension, ARDS, and hypoxic respiratory failure in children (54–56). However, unlike corticosteroids and NMBAs, using iNO in ARDS has been highly controversial. In 2007, Adhikari et al. reported that iNO was associated with renal insufficiency in ARDS patients, with limited metabolic improvements and potential harm (57). A follow-up study in 2014 (58) reached similar conclusions, showing that iNO does not reduce mortality in adults or children with ARDS. Consistent with these findings, our NMA demonstrated that iNO does not improve mortality rates or ventilator-free days in ARDS patients. Several factors may explain why iNO fails to improve outcomes. First, the prolonged fixed-dosing regimens used in most trials may diminish their benefits over time, as increased sensitivity can inhibit oxygenation improvements while exposing patients to potential toxic effects, such as oxidative damage (59). Second, even in severe cases of hypoxemia, ARDS patients often succumb to primary respiratory failure rather than multiple organ failure. In such cases, the modest oxygenation benefits of iNO may be outweighed by the harmful effects of mechanical ventilation strategies employed in most trials, which often lack strict limitations on tidal volume or airway pressure.

### Strengths and limitations of the study

This NMA is the first to directly compare the effectiveness and safety of three treatments for ARDS patients. Building on prior research, we included new and relevant studies while excluding low-quality or ineligible ones, aiming to provide a robust evidence base for ARDS treatment. However, several limitations, including potential confounders and variability in study quality, warrant a detailed discussion.

The quality of the included studies varied which may have influenced the results. While most included studies were RCTs, a few non-RCTs were included to ensure comprehensive data coverage. Non-RCTs, lacking methodological rigor such as randomization and blinding, are prone to selection, performance, and detection biases. For example, open-label designs may introduce observer bias, particularly in subjective outcomes like infection rates or ventilator-free days. Additionally, inconsistencies in trial protocols, including randomization methods, blinding, and follow-up duration, further complicate result interpretation. Double-blind studies minimize bias, but unblinded designs may inflate perceived treatment effects. Shorter follow-up periods may underestimate adverse effects or fail to capture long-term treatment benefits.

Including studies with varying designs, such as observational and non-RCTs, introduces additional complexity. Observational studies, while valuable for hypothesis generation, are prone to confounding due to the absence of randomization. Variations in baseline characteristics, such as ARDS severity, comorbidities, and treatment settings, may disproportionately influence outcomes, particularly in studies with smaller sample sizes. Although network meta-analyses accommodate data from diverse study designs, including heterogeneous designs raises concerns about comparing populations, interventions, and outcomes. Such heterogeneity may contribute to variations in effect sizes and confidence intervals, potentially limiting the generalizability of findings.

Despite efforts to control for confounders, residual confounding likely influenced the results. Differences in baseline patient characteristics, such as age, sex, comorbidities, and immune status, may affect treatment efficacy and safety. For example, younger ARDS patients often exhibit better immune resilience, potentially amplifying the perceived benefits of corticosteroids or neuromuscular blocking agents in studies involving younger populations. Similarly, comorbidities such as diabetes or chronic kidney disease may impact outcomes such as infection rates or mortality. Geographical differences and variations in healthcare systems further contribute to confounding factors. Patients in countries with advanced critical care infrastructure may experience better outcomes irrespective of the intervention, due to higher baseline care quality. For instance, studies conducted in high-income countries may report lower mortality rates than those from low- or middle-income countries, reflecting differences in supportive care rather than the effectiveness of interventions themselves.

Variations in patient recruitment criteria, particularly in PaO₂/FiO₂ thresholds, introduced heterogeneity in ARDS severity among included studies. Some trials exclusively enrolled patients with severe ARDS, while others included a broader spectrum of disease severity. These differences may skew the results, as treatments exhibit varying efficacy across different ARDS severities. For instance, Herwig Gerlach’s study focused on severe ARDS, potentially overstating the benefits of treatments that may not perform as effectively in milder cases.

Our analysis of adverse effects was limited to new infections, and infection definitions varied across studies. This inconsistency may have introduced additional bias and underestimated other adverse events, such as renal insufficiency or neuromuscular complications. Furthermore, inconsistent reporting of adverse effects across trials restricted the evaluation of comprehensive safety profiles for each intervention.

This study primarily evaluated short-term outcomes, such as 28-day mortality and ventilator-free days. Still, it did not assess long-term outcomes such as functional recovery, quality of life, or survival beyond 28 days. While short-term metrics are relevant for acute interventions, they do not capture the full impact of treatments on patient outcomes. Future studies should explore long-term implications, particularly as ARDS survivors often face prolonged morbidity and impaired lung function.

To address these limitations, future studies should focus on well-designed, high-quality RCTs with consistent recruitment criteria, rigorous randomization, and standardized outcome reporting, covering both short- and long-term metrics. Meta-analyses of individual patient data (IPD) could provide more granular insights by enabling subgroup analyses based on age, severity, and comorbidities. Additionally, further research is needed to comprehensively evaluate the safety profiles of the interventions, particularly for rare but serious adverse events, and to better understand their long-term effects.

## Conclusion

This NMA indicates that corticosteroids improve short-term survival, increase ventilator-free days, and reduce infection rates in patients with ARDS. NMBAs may also minimize 28-day mortality, while iNO offers no significant benefit. Based on these findings, we recommend the use of corticosteroids or NMBAs in the treatment of ARDS. However, important questions remain, including the long-term survival benefits, variations in treatment efficacy based on the underlying cause of ARDS, and optimal protocols for the type, dosage, and duration of corticosteroid or NMBA use. Future trials should address these critical gaps to refine treatment strategies further.

## Data Availability

The original contributions presented in the study are included in the article/Supplementary material, further inquiries can be directed to the corresponding authors.
