# Supplementary material for: Comparative outcomes of corticosteroids, neuromuscular blocking agents, and inhaled nitric oxide in ARDS: a systematic review and network meta-analysis
Source: Front Med (Lausanne). 2025 Feb 3;12:1507805. doi: 10.3389/fmed.2025.1507805 (PMC11831700; doi:10.3389/fmed.2025.1507805)
Supplement: Supplementary file 3 [file Data_Sheet_3.docx]

**Supplemental Online Content**

**eAppendix 1.** Search Strategy

**eFigure 1.** Risk of Bias of Included Studies

**eFigure 2.** Funnel Plots for outcomes

**eTable 1.** Consistency and Heterogeneity Analysis

**eTable 2.** Node Splitting of In-hospital Mortality

**eTable 3.** Node Splitting of New infection events

**eAppendix 1.** Search Strategy

**MEDLINE (via PubMed)**

1 "Respiratory Distress Syndrome"[Mesh]

2 "acute respiratory distress syndrome"[Title/Abstract] OR "distress syndrome respiratory"[Title/Abstract] OR "distress syndromes respiratory"[Title/Abstract] OR "respiratory distress syndromes"[Title/Abstract] OR "syndrome respiratory distress"[Title/Abstract] OR "shock lung"[Title/Abstract] OR "ARDS"[Title/Abstract]

3 #1 OR #2

4 "Adrenal Cortex Hormones"[MeSH Terms]

5 "Corticosteroids"[Title/Abstract] OR "hormones adrenal cortex"[Title/Abstract] OR "Corticosteroid"[Title/Abstract] OR "Corticoids"[Title/Abstract] OR "Corticoid"[Title/Abstract] OR "adrenal cortex hormone"[Title/Abstract] OR (("Cortex"[All Fields] OR "cortex s"[All Fields] OR "cortexes"[All Fields]) AND "hormone adrenal"[Title/Abstract]) OR "hormone adrenal cortex"[Title/Abstract]

6 #4 OR #5

7 #3 AND #6

8 "Nitric Oxide"[MeSH Terms]

9 "nitrogen monoxide"[Title/Abstract] OR "monoxide nitrogen"[Title/Abstract] OR "endogenous nitrate vasodilator"[Title/Abstract] OR ((("nitratation"[All Fields] OR "nitrates"[MeSH Terms] OR "nitrates"[All Fields] OR "Nitrate"[All Fields]) AND ("vasodilate"[All Fields] OR "vasodilated"[All Fields] OR "vasodilates"[All Fields] OR "vasodilating"[All Fields] OR "vasodilation"[MeSH Terms] OR "vasodilation"[All Fields] OR "vasodilations"[All Fields] OR "vasodilative"[All Fields] OR "vasodilator agents"[Pharmacological Action] OR "vasodilator agents"[MeSH Terms] OR ("Vasodilator"[All Fields] AND "agents"[All Fields]) OR "vasodilator agents"[All Fields] OR "Vasodilator"[All Fields] OR "vasodilators"[All Fields])) AND "Endogenous"[Title/Abstract]) OR (("vasodilate"[All Fields] OR "vasodilated"[All Fields] OR "vasodilates"[All Fields] OR "vasodilating"[All Fields] OR "vasodilation"[MeSH Terms] OR "vasodilation"[All Fields] OR "vasodilations"[All Fields] OR "vasodilative"[All Fields] OR "vasodilator agents"[Pharmacological Action] OR "vasodilator agents"[MeSH Terms] OR ("Vasodilator"[All Fields] AND "agents"[All Fields]) OR "vasodilator agents"[All Fields] OR "Vasodilator"[All Fields] OR "vasodilators"[All Fields]) AND "endogenous nitrate"[Title/Abstract]) OR "mononitrogen monoxide"[Title/Abstract] OR "iNO"[Title/Abstract]

10 #8 OR #9

11 #3 AND #10

12 "Neuromuscular Blocking Agents"[MeSH Terms]

13 "agents neuromuscular blocking"[Title/Abstract] OR (("block"[All Fields] OR "blocked"[All Fields] OR "Blocking"[All Fields] OR "blockings"[All Fields] OR "blocks"[All Fields]) AND "agents neuromuscular"[Title/Abstract]) OR "neuromuscular blockers"[Title/Abstract] OR "blockers neuromuscular"[Title/Abstract] OR "neuromuscular blocker"[Title/Abstract] OR "blocker neuromuscular"[Title/Abstract] OR "neuromuscular blocking agent"[Title/Abstract] OR "agent neuromuscular blocking"[Title/Abstract] OR "blocking agent neuromuscular"[Title/Abstract]

14 #12 OR #13

15 #3 AND #14

**EMBASE (via Embase.com)**

#1. ('respiratory'/exp OR respiratory) AND ('distress'/exp OR distress) AND ('syndrome'/exp OR syndrome)

#2. 'respiratory distress syndrome':ab,ti OR 'acute respiratory distress syndrome':ab,ti OR 'distress syndrome, respiratory':ab,ti OR 'distress syndromes, respiratory':ab,ti OR 'respiratory distress syndromes':ab,ti OR 'syndrome, respiratory distress':ab,ti OR 'shock lung':ab,ti OR 'ards':ab,ti

#3. #1 OR #2

#4. 'adrenal cortex hormones therapy'

#5. 'corticosteroids':ab,ti OR 'hormones,adrenal cortex':ab,ti OR 'corticosteroid':ab,ti OR 'corticoids':ab,ti OR 'corticoid':ab,ti OR 'adrenal cortex hormone':ab,ti OR 'cortex hormone, adrenal':ab,ti OR 'hormone, adrenal cortex':ab,ti

#6. #4 OR #5

#7. #3 AND #6

#8. 'nitric oxide'

#9. 'nitrogen monoxide':ab,ti OR 'monoxide,nitrogen':ab,ti OR 'endogenous nitrate vasodilator':ab,ti OR 'nitrate vasodilator,endogenous':ab,ti OR 'vasodilator, endogenous nitrate':ab,ti OR 'mononitrogen monoxide':ab,ti OR 'ino':ab,ti

#10. #8 OR #9

#11. #3 AND #10

#12. 'neuromuscular blocking agent'

#13. 'agents, neuromuscular blocking':ab,ti OR 'blocking agents, neuromuscular':ab,ti OR 'neuromuscular blockers':ab,ti OR 'blockers,neuromuscular':ab,ti OR 'neuromuscular blocker':ab,ti OR 'blocker, neuromuscular':ab,ti OR 'neuromuscular blocking agent':ab,ti OR 'agent, neuromuscular blocking':ab,ti OR 'blocking agent, neuromuscular':ab,ti

#14. #12 OR #13

#15. #3 AND #14

**COCHRANE (via Cochrane library)**

#1 Respiratory Distress Syndrome

#2 (Acute Respiratory Distress Syndrome):ab,ti,kw OR (Distress Syndrome, Respiratory):ab,ti,kw OR (Distress Syndromes, Respiratory):ab,ti,kw OR (Respiratory Distress Syndromes):ab,ti,kw OR (Syndrome, Respiratory Distress):ab,ti,kw OR (Shock Lung):ab,ti,kw OR (ARDS):ab,ti,kw

#3 #1 OR #2

#4 Adrenal Cortex Hormones

#5 (Corticosteroids):ab,ti,kw OR (Hormones,Adrenal Cortex):ab,ti,kw OR (Corticosteroid):ab,ti,kw OR (Corticoids ):ab,ti,kw OR (Corticoid ):ab,ti,kw OR (Adrenal Cortex Hormone ):ab,ti,kw OR (Cortex Hormone, Adrenal ):ab,ti,kw OR (Hormone, Adrenal Cortex):ab,ti,kw

#6 #4 OR #5

#7 #3 AND #6

#8 Oxide, Nitric

#9 (Nitrogen Monoxide):ab,ti,kw OR (Monoxide, Nitrogen):ab,ti,kw OR (Endogenous Nitrate Vasodilator):ab,ti,kw OR (Nitrate Vasodilator, Endogenous):ab,ti,kw OR (Vasodilator, Endogenous Nitrate):ab,ti,kw OR (Mononitrogen Monoxide):ab,ti,kw OR (iNO):ab,ti,kw

#10 #8 OR #9

#11 #3 AND #10

#12 Neuromuscular Blocking Agents

#13 (Agents, Neuromuscular Blocking):ab,ti,kw OR (Blocking Agents, Neuromuscular):ab,ti,kw OR (Neuromuscular Blockers):ab,ti,kw OR (Blockers, Neuromuscular):ab,ti,kw OR (Neuromuscular Blocker):ab,ti,kw OR (Blocker, Neuromuscular):ab,ti,kw OR (Neuromuscular Blocking Agent):ab,ti,kw OR (Agent, Neuromuscular Blocking):ab,ti,kw OR (Blocking Agent, Neuromuscular):ab,ti,kw

#14 #12 OR #13

#15 #3 AND #14

**WEB OF SCIENCE (via Web of science)**

1: TS=(Corticosteroids OR Adrenal Cortex Hormones OR Corticosteroids OR Hormones,Adrenal Cortex OR Corticosteroid OR Corticoids OR Corticoid OR Adrenal Cortex Hormone OR Cortex Hormone, Adrenal OR Hormone, Adrenal Cortex )

2: TS=(Respiratory Distress Syndrome OR Acute Respiratory Distress Syndrome OR Distress Syndrome, Respiratory OR Distress Syndromes, Respiratory OR Respiratory Distress Syndromes OR Syndrome, Respiratory Distress OR Shock Lung OR ARDS)

3: #1 AND #2

4:TS=(Oxide, Nitric OR Nitrogen Monoxide OR Monoxide, Nitrogen OR Endogenous Nitrate Vasodilator OR Nitrate Vasodilator, Endogenous OR Vasodilator, Endogenous Nitrate OR Mononitrogen Monoxide OR iNO)

5: #2 AND #4

6: TS=(Neuromuscular Blocking Agents OR Agents, Neuromuscular Blocking OR Blocking Agents, Neuromuscular OR Neuromuscular Blockers OR Blockers, Neuromuscular OR Neuromuscular Blocker OR Blocker, Neuromuscular OR Neuromuscular Blocking Agent OR Agent, Neuromuscular Blocking OR Blocking Agent, Neuromuscular)

7: #2 AND #6

**eFigure 1**. Risk of Bias of Included Studies

**Risk of bias graph**


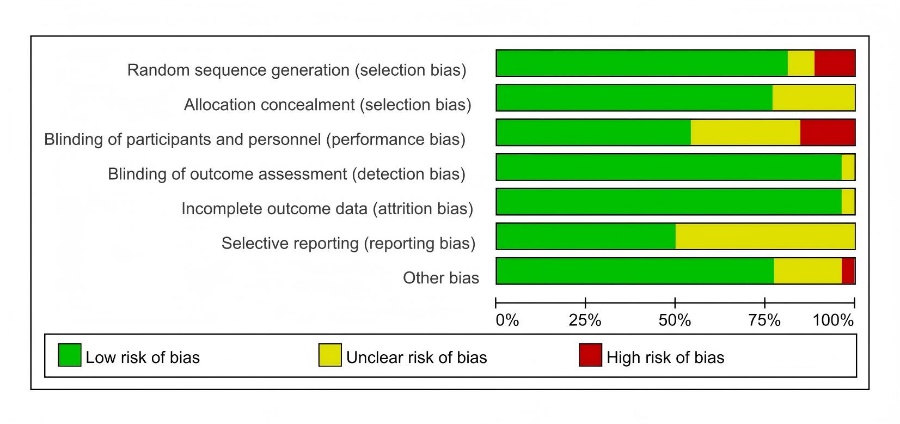


**Risk of bias summary**


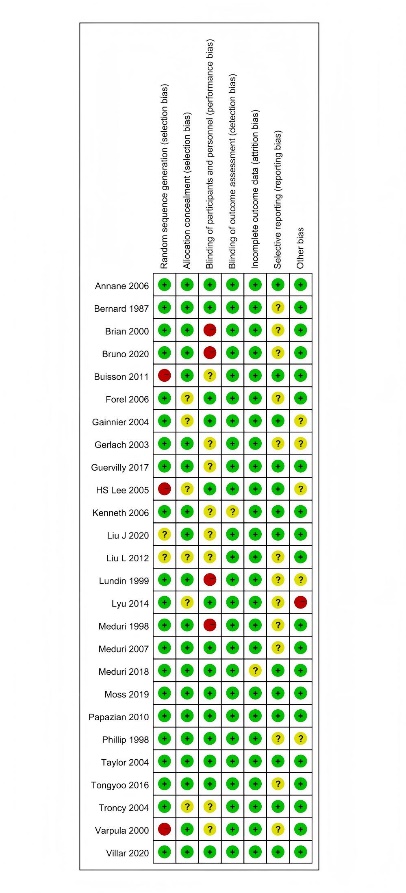


Overall, 5 trials were judged to have a low risk of bias across all domains.

3 trials were judged to have a high risk of bias for random sequence generation and 4 trials were judged to have a high risk of bias for blinding of participants and personnel due to inadequate randomization and assignments.

Most studies were judged to have a low risk of bias for incomplete outcome data and blinding of outcome assessment; however, 1 trial was judged to have a high risk of bias for other bias, as small sample size and loss to follow-up could have a clinically relevant impact on the intervention effect estimates.

**eFigure 2.** Funnel Plots for outcomes


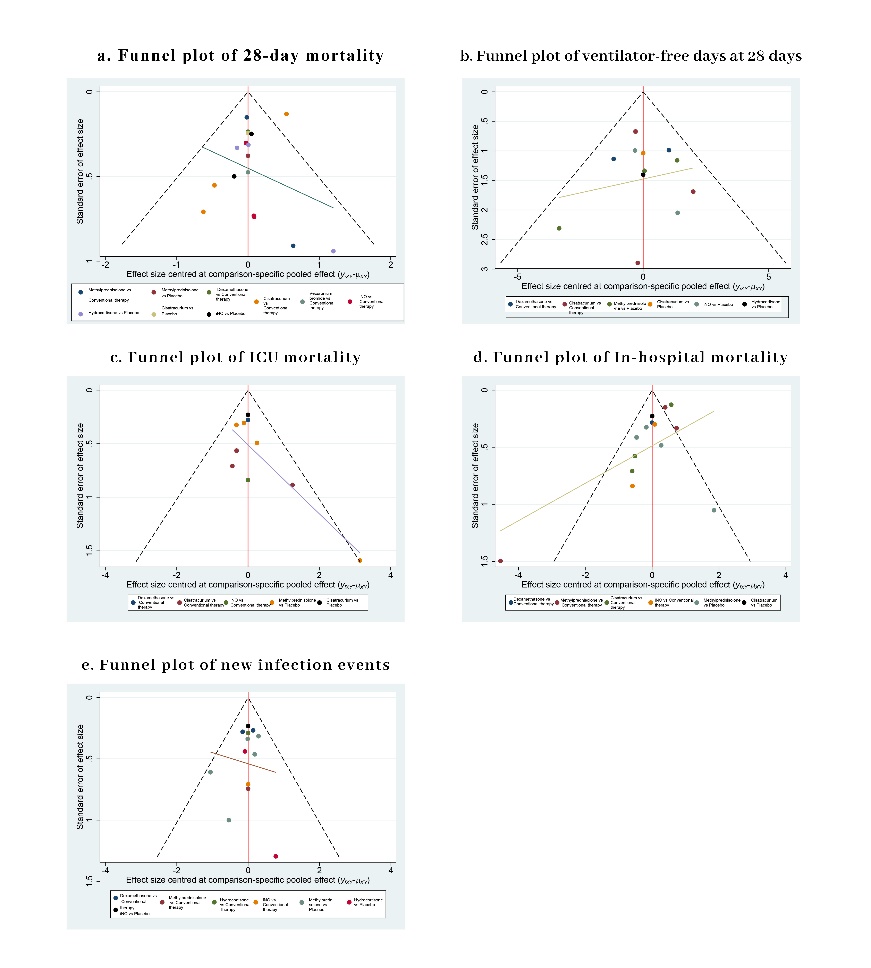


**Note:** The vertical axis represents the standard error (SE) of the effect size estimates

The diagonal line intersecting with the red vertical line**:** Represents the fitted regression line for asymmetry assessment.

The red vertical line**:** Represents the line of no effect (OR = 1 or SD = 0).

**eTable 1.** Consistency and Heterogeneity Analysis

| **Outcome** | **Chi²** | **P** | **τ²** |
| --- | --- | --- | --- |
| **Ventilator-free days at 28 days** | 0.22 | 0.6373 | 0.00602197 |
| **28-day mortality** | 2.03 | 0.3615 | 0.03968078 |
| **ICU mortality** | 3.40 | 0.0653 | 0.02571854 |
| **In-hospital mortality** | 1.74 | 0.1875 | 0.30297074 |
| **New infection events** | 2.10 | 0.3492 | 0.07728789 |

**eTable 2. Node Splitting of In-hospital Mortality**

| **In-hospital mortality** | **Direct** | | **Indirect** | | **Difference** | | **P>\|z\|** |
| --- | --- | --- | --- | --- | --- | --- | --- |
|  | **Coef.** | **Std. Err.** | **Coef.** | **Std. Err.** | **Coef.** | **Std. Err.** |  |
| 1:2 | 0.6766117 | 0.7240547 | -0.0318941 | 186.917 | 0.7085058 | 186.9184 | 0.997 |
| 2:3 | 0.3942464 | 0.5333305 | -0.8092196 | 0.8937469 | 1.203466 | 0.9130567 | 0.187 |
| 2:5 | -0.5421428 | 0.4701999 | 0.6613143 | 0.839241 | -1.203457 | 0.9130597 | 0.187 |
| 2:6 | 0.1072529 | 0.6077466 | -1.355192 | 1000.103 | 1.462445 | 1000.103 | 0.999 |
| 3:4 | 0.6623327 | 0.3994168 | -0.5411329 | 0.8391831 | 1.203466 | 0.9130603 | 0.187 |
| 4:5 | -0.3952562 | 0.5952333 | -1.598718 | 0.6923727 | 1.203461 | 0.9130621 | 0.187 |

**Note**:

1: Dexamethasone; 2: Conventional therapy; 3: Dexamethasone; 4: Placebo; 5: Cisatracurium;

6: iNO

**eTable 3.** Node Splitting of New infection events

| **New infection events** | **Direct** | | **Indirect** | | **Difference** | | **P>\|z\|** |
| --- | --- | --- | --- | --- | --- | --- | --- |
|  | **Coef.** | **Std. Err.** | **Coef.** | **Std. Err.** | **Coef.** | **Std. Err.** |  |
| 1:2 | 0.2408296 | 0.1931938 | -0.3050985 | 227.0315 | 0.5459281 | 227.0316 | 0.998 |
| 2:3 | 0.9444616 | 0.7442904 | -0.3215647 | 0.4606433 | 1.266026 | 0.875306 | 0.148 |
| 2:5 | 0.4411832 | 0.2891556 | 0.9864922 | 0.6773629 | -0.545309 | 0.7364995 | 0.459 |
| 2:6 | 0.8237673 | 0.706911 | 1.297891 | 0.4826483 | -0.4741237 | 0.8559629 | 0.58 |
| 3:4 | 0.550272 | 0.1919664 | -0.7157524 | 0.853996 | 1.266024 | 0.8753058 | 0.148 |
| 4:5 | 0.1806815 | 0.4158985 | -0.3646287 | 0.6078325 | 0.5453102 | 0.7364998 | 0.459 |
| 4:6 | 0.6636813 | 0.2325021 | 0.189557 | 0.8237811 | 0.4741243 | 0.8559629 | 0.58 |

**Note**:

1: Dexamethasone; 2: Conventional therapy; 3: Methylprednisolone; 4: Placebo; 5: Hydrocortisone; 6: iNO
